# Are enlarged peroneal tubercle and accessory anterolateral talar facet associated with calcaneal spur?

**DOI:** 10.1186/s13018-024-04718-8

**Published:** 2024-04-12

**Authors:** Yuichi Kasai, Permsak Paholpak, Taweechok Wisanuyotin, Nuttharada Sukitthanakornkul, Parika Hanarwut, Arada Chaiyamoon, Sitthichai Iamsaard, Akinobu Nishimura

**Affiliations:** 1 Department of Orthopaedics, Faculty of Medicine, Khon Kaen University, 123 Moo 16 Mittraphap Rd., Nai-Muang, Muang District, Khon Kaen, 40002 Thailand; 2 Department of Anatomy, Faculty of Medicine, Khon Kaen University, Khon Kaen, Thailand; 3 Department of Orthopaedic and Sports Medicine, Graduate School of Medicine, Mie University, Tsu, Japan

**Keywords:** Foot, Anatomical variations, Peroneal tubercle, Accessory anterolateral talar facet, Calcaneal spur

## Abstract

**Background:**

As the anatomical variations of the foot, enlarged peroneal tubercle (EPT) and accessory anterolateral talar facet (AALTF) have attracted the attention of foot surgeons in recent years. However, EPT and AALTF have not been examined for a relationship with calcaneus spur (CS) as a common osteophyte.

**Methods:**

The subjects were 369 individuals who died in northeastern Thailand and were preserved as skeletal specimens. The authors examined for the presence of left and right EPT, AALTF, and calcaneus spur (CS). We divided the EPT (+) group with EPT and the EPT (-) group without it and also divided the AALTF (+) group with AALTF and the AALTF (-) group without it. The age at death and the presence of CS were compared statistically between the EPT (+) and EPT (-) groups and between the AATLF (+) and AALTF (-) groups.

**Results:**

Out of the total 369 cases, EPT was found in 117 cases (31.7%), AALTF was positive in 91 cases (24.7%), and CS was found in 194 cases (52.3%). In comparison between EPT (+) and EPT (-) groups, CS was significantly higher (*p* < 0.0001) in the EPT (+) group, but there was no significant difference in age at death. In comparison between AALTF (+) and AALTF (-) groups, there was no significant difference in age at death or CS.

**Conclusion:**

This study showed a strong relationship between EPT and CS, and the prevalence of EPT and AALTF by age in Thailand was first reported. We believe it helps to know the pathogenesis and biomechanism of EPT and AALTF.

**Trial registration:**

Not applicable.

## Background

**Fig. 1:**
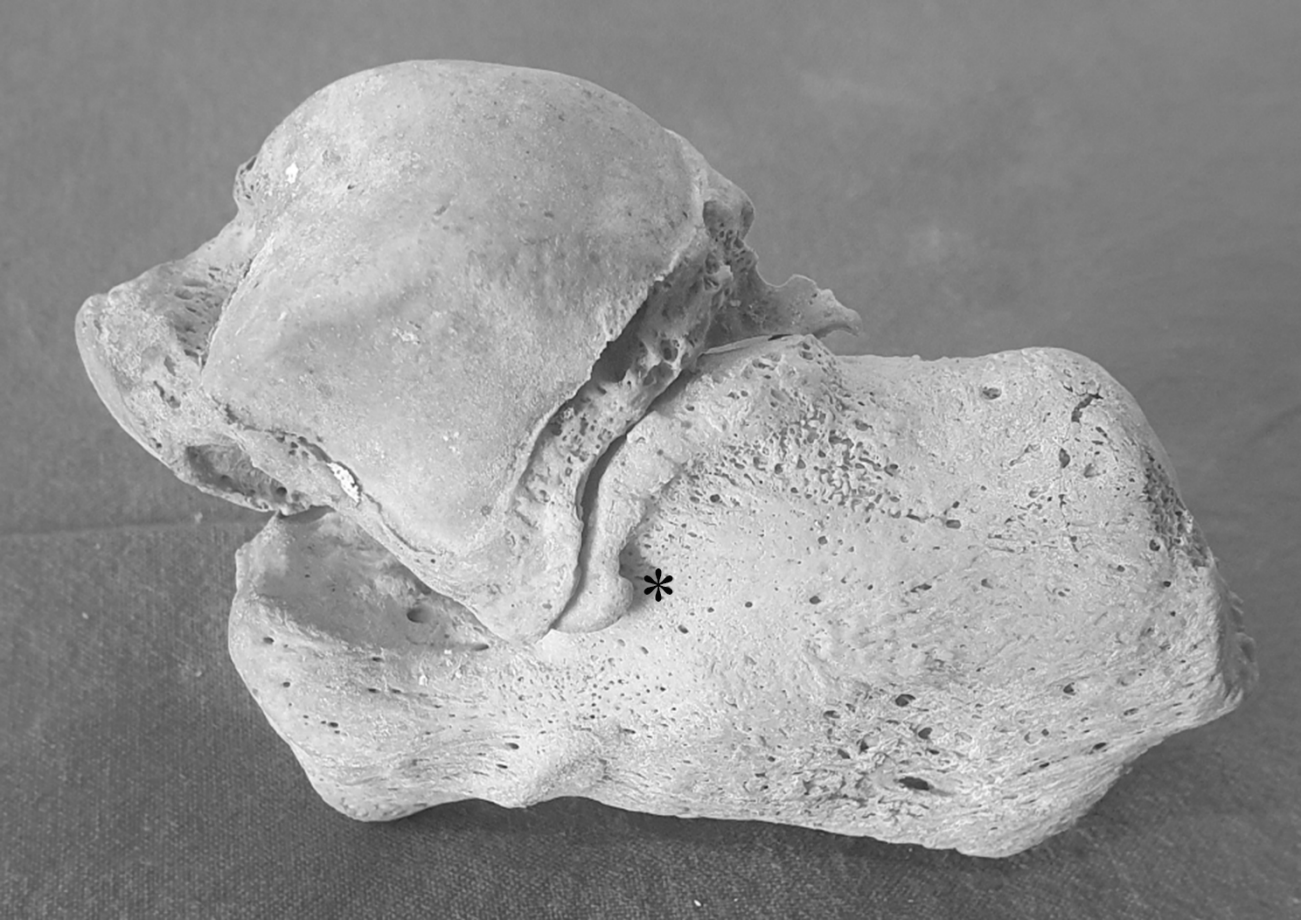
The dry bone of talus and calcaneus. The asterisk indicates the Accessory anterolateral talar facet (AALTF)

**Fig. 2:**
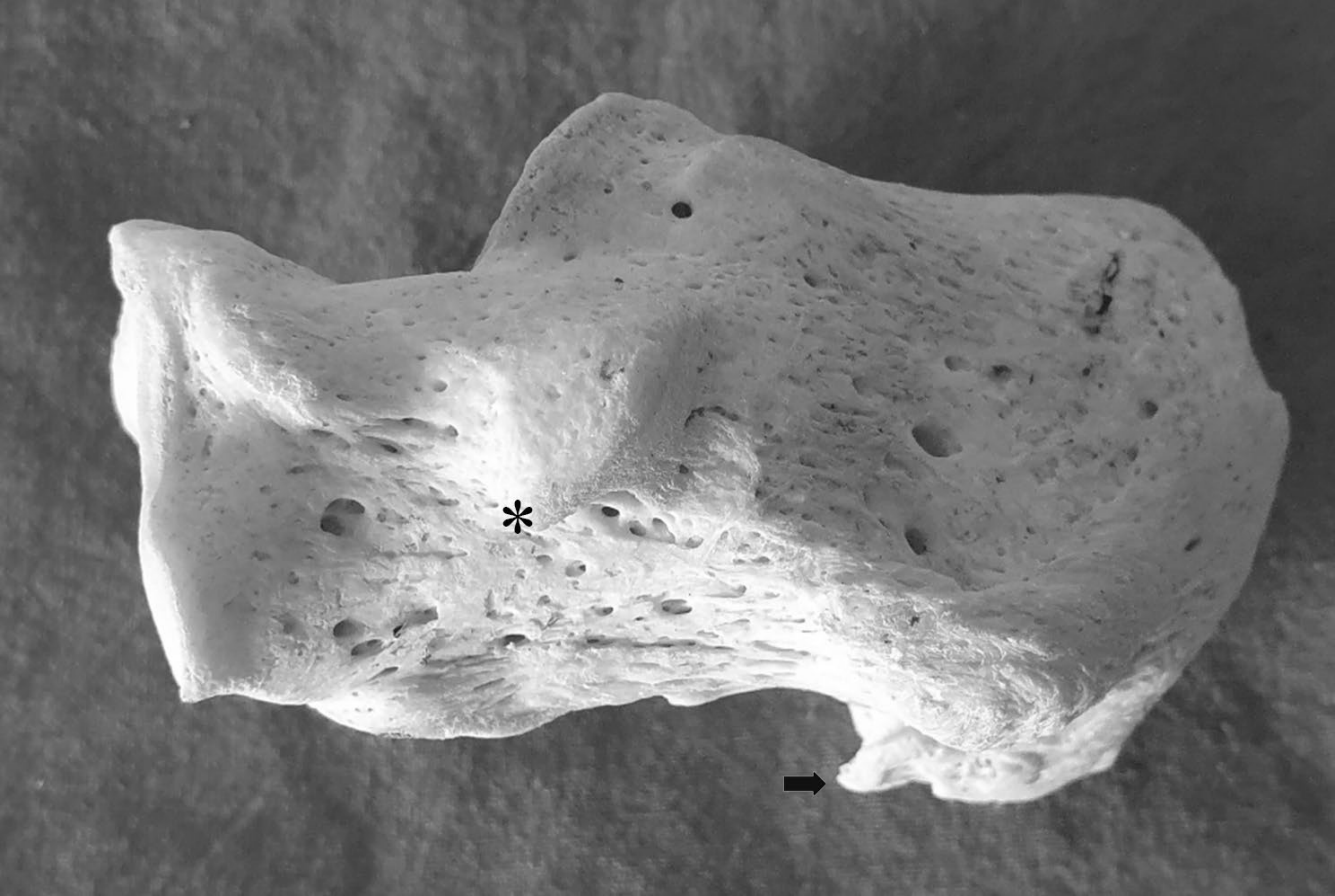
The dry bone of calcaneus. The asterisk indicates the Enlarged peroneal tubercle (EPT) and the arrow indicates the calcaneal spur (CS)

Peroneal tubercle (PT) was reported by Laidlaw [1] in 1904, and accessory anterolateral talar facet (AALTF, Fig. 1) was written by Sewell [2] in 1904. However, initially, because of their less clinical significance, no relevant studies were conducted on PT and AALTF. However, in 1992, Pierson et al. [3] reported a case of stenosing peroneal tenosynovitis caused by enlarged peroneal tubercle (EPT, Fig. 2), and in 2008, Martus et al. [4, 5] reported cases of impingement between the talus and the calcaneus by AALTF, and since then EPT and AALTF have come to the attention of foot surgeons.

Recent osteogenic studies showed that the size of PT is significantly correlated with the size of the lateral plantar process, the volume of the retrotrochlear eminence, and the height of the navicular [6]. It has been reported that AALTF is significantly related to tarsal coalitions such as intra-articular middle facet talocalcaneal coalition and extra-articular posteromedial talocalcaneal coalition [7]. However, the relationship between EPT or AALTF and calcaneus spur (CS, Fig. 2), which exists a little away from them, have not been investigated. Therefore, we were the first to report the prevalence of EPT and AALTF in Thailand, explored the relationship between them and CS, and discussed the pathogenesis or biomechanism of EPT and AALTF.

## Materials and methods

The materials were the whole body’s dry bones of 394 cases who died in northeastern Thailand, and they were donated to the Department of Anatomy at the Khon Kaen University and checked by the University’s Unit of Human Bone Warehouse for Research. Among them, the dry bones of 369 cases in which both talus and calcaneus were present and were measurable without obvious fractures or surgical damages were selected and investigated. The age at the time of death was 23 to 93 years old (average 67.7 years old), and 332 samples were of men and 37 of women.

The items investigated were the presence or absence of EPT and AALTF, and the presence or absence of left and right calcaneus spurs (CS). PT and calcaneus spur size were measured using a digital vernier caliper (Mitutoyo series 500 − 193 ABSOLUTE Digimatic Caliper, 300 mm/12”, Japan). The EPT was determined positive if there was a protrusion of 5 mm or more according to the criteria of Vosoughi et al. and Lui et al. [8, 9]. To determine AALTF, it was positive if there was a clear facet formation macroscopically on the anterolateral joint surface of the talus and calcaneus. Regarding CS, based on the measured values of Kuyucu et al. and Kirkpatrick et al. [10, 11], it was positive if there was an apparent protrusion of 3 mm or more.

Three evaluators (orthopaedic surgeons) decided on the presence or absence of EPT, AALTF, and CS. Evaluator 1 (YK) took measurements twice, and Evaluator 2 (PP) took measurements once. Evaluator 1’s first and second measurements and Evaluator 2’s measurements were conducted separately with an interval of one week or more between each. These three data were then entered into separate EXCEL sheets. If the total three evaluations of evaluator 1, the first, second, and evaluator 2, were different, a third evaluator (TW) measured at one week or more intervals, and the results were entered into completely different EXCEL sheets.

We categorized the cases with EPT as EPT (+) group, the cases without EPT as EPT (-) group, and we performed the comparative studies between two groups. We also regarded the cases with AALTF as AALTF (+) group and the cases without AALTF as AALTF (-) group, and we carried out the comparative studies between two groups. As for the statistical analysis, the Mann-Whitney U test was used for age, and Fisher’s exact test was used for the presence or absence of CS, and *p* < 0.05 was determined to be a significant difference. Inter-observer agreement between the two evaluators (YK and PP), and intra-observer agreement by one evaluator (YK) were analyzed using kappa statistics for EPT, AALTF, and CS. The inter-observer agreement between the two evaluators was analyzed using data from evaluator 2’s and evaluator 1’s first measurements. The kappa and ICC value was assessed as follow: 0–0.2 indicated slight agreement, 0.21–0.4 fair agreement, 0.41–0.6 moderate agreement, 0.61–0.8 substantial agreement, and 0.81–1 excellent agreement. The level of significance (p value) was set at 0.05. This study was approved by the Ethics Committee of the Khon Kaen University in Thailand (Approval Number HE611293).

## Results

Out of the total 369 cases, EPT was found in 117 cases (31.7%, 67 cases on both sides and 50 cases on one side), and AALTF was positive in 91 cases (24.7%, 45 cases on both sides and 46 cases on one side), and the rate of presence by the age and sex of EPT and AALTF is shown in Table 1.

**Table 1:** Presence of EPT and AALTF by age group and sex

	20s	30s	40s	50s	60s	70s	80s	90s
No. of male cases (332 cases)	1	3	30	55	95	97	44	7
EPT (+) cases (103 cases)	0	0	10 (33.3%)	15 (27.3%)	28 (29.5%)	33 (34.0%)	14 (31.8%)	3 (42.9%)
AATLF (+) cases (76 cases)	0	0	5 (16.7%)	11 (20.0%)	28 (29.5%)	21 (21.6%)	8 (18.2%)	3 (42.9%)
No. of female cases (37 cases)	0	1	3	7	8	12	6	0
EPT (+) cases (14 cases)	0	0	1	3	1	6	3	0
AATLF (+) cases (15 cases)	0	1	3	1	2	6	2	0

EPT: Enlarged peroneal tubercle, AALTF: accessory anterolateral talar facet

According to the comparative results between two groups (Table 2), the EPT (+) group (117 cases, 103 men, 14 women) had an average age of 68.1 years and 89 positive CS cases (62 on both sides, ten on the right, 17 on the left). Whereas EPT (-) group (252 cases, 229 cases of men, 23 cases of women) had an average age of 66.0 years, and 105 positive CS cases (78 cases on both sides, 13 cases on the right side, 14 cases on the left side). The AALTF (+) group (91 cases, 76 men, 15 women) had an average age of 66.6 years and 48 CS positive cases (37 on both sides, three on the right, eight on the left), and whereas AALTF (-) group (278 cases, 256 cases of men, 22 cases of women) had an average age of 66.7 years and 146 CS positive cases (103 cases on both sides, 20 cases on the right side, 23 cases on the left side). The kappa values for EPT were 0.877 (95% CI: 0.837–0.917) for inter-observer and 0.891 (95% CI: 0.853–0.929) for intra-observer. The kappa values for AALTF were 0.820 (95% CI: 0.766–0.874) for inter-observer and 0.847 (95% CI: 0.797–0.897) for intra-observer. The kappa values for CS were 0.891 (95% CI: 0.858–0.924) for inter-observer and 0.904 (95% CI: 0.873–0.935) for intra-observer.

**Table 2:** Age and the number of CS (+) cases in comparison between EPT (+) and EPT (-) groups or AALTF (+) and AALTF (-) groups

	EPT (+) 117 cases (103 men, 14 women)	EPT (-) 252 cases (229 men, 23 women)	AALTF (+) 91 cases (76 men, 15 women)	AALTF (-) 278 cases (256 men, 22 women)
CS (+)	89 cases (76.1%) * 62 on both sides 10 on the right 17 on the left	105 cases (41.7%) * 78 on both sides 13 on the right 14 on the left	48 cases (52.7%) 37 on both sides 3 on the right 8 on the left	146 cases (52.5%) 103 on both sides 20 on the right 23 on the left
CS (-)	28 (23.9%)	147 (58.3%)	43 (47.3%)	132 (47.5%)
age	68.1 ± 10.4 years old (mean ± SD)	66.0 ± 12.6 years old (mean ± SD)	66.6 ± 12.4 years old (mean ± SD)	66.7 ± 12.7 years old (mean ± SD)

* Significant presence (*p* < 0.0001) of CS between the EPT (+) group and EPT (-) group EPT: Enlarged peroneal tubercle, AALTF: accessory anterolateral talar facet, CS: Calcaneal spur, SD: Standard deviation

In the statistical analysis of 90 cases between the two groups on EPT, there was a significant rate of presence (*p* < 0.0001) in CS in the EPT (+) group, but there was no significant difference in age. Regarding AALTF, there was no significant difference between the two groups in both age and CS.

## Discussion

EPT may lead to the degeneration and damage of peroneal tendons and the appearance of stenosing peroneal tenosynovitis. It is an important disease in tendon disorders because EPT causes lateral foot pain, clicking sensation, discomfort when wearing shoes, and limitation of gait [12–15]. EP itself is reported to be found in 90% of people [16, 17], and EPT is found in 20–44% [18, 19]. The rate of presence reported in this study was 31.6%, which was within the range previously reported, and the prevalence was about 30% for those over 40 years old by age group. Taneja et al. [13] and Shibata et al. [20] reported that EPT was slightly more in men and is increasingly found in middle-aged or older age groups than in younger people, but our study found less data on young people and women, which could not be concluded.

AALTF is a congenital variance, which forms a facet between the anterolateral talocalcaneal joint, the average facet size is 7 mm, that causes impingement between talus and calcaneus, resulting in pain in the lateral part of the ankle joint [4, 21, 22]. It was reported that the prevalence of AALTF was 3.6–32.7% [23, 24], and there was a report that it was also seen in 34% of children [5]. The rate of presence on AALTF in our study was 24.7%, which was within the range previously reported, and the prevalence might be about 20% for those over 40 years old by age group. Although the high prevalence has been reported in men [5, 8], it could not be concluded because there is little data available on women in the present study.

Here, we would also like to consider the pathogenesis of EPT and AALTF a little. First of all, because CS is thought to be caused by aging, obesity, physical activity, hard labor, leg deformity, the presence of arthritis [11, 25, 26], people with CS (+) might have had a lot of mechanical loads to the plantar region. From the results of our study that CS was significantly seen in the EPT (+) group, it is inferred that there is a more mechanical load on the sole of the people with EPT than in those of AALTF. We guess that tightness occurs in the peroneal tendons in people with EPT by aging, and mechanical stimulation or load is gradually added to the peroneal tubercle and made the larger size of EPT, and then, clinical symptoms such as pain and discomfort might appear. There have been some reports of flat foot accompanied with AALTF [4, 22, 27], however, because the present study showed that there was no significant relationship between CS that is deeply related to flat foot and AALTF, it was speculated that AALTF might not have occurred due to talocalcaneal impinge by flat foot. Therefore, both EPT and AALTF are congenital variants, however, EPT might be more susceptible to various acquired influences by the mechanical loads or mechanical stimulations to the plantar region than AALTF.

The limitations of this study were as follow: (1) Clinical information such as body weight, occupation, and foot pain was not obtained at all except for age and sex, (2) Gender differences could not be examined because there were few female samples, (3) We set the CS cutoff value to 3 mm, but this value is controversial, (4) The cartilage lesion could not be evaluated for observation of dried bone in the present study, and (5) The number of samples might still not be enough to obtain the prevalence of EPT and AALTF. As the above described, there are many limitations because of the research with cadaveric bones, but we would like to conduct further research in a clinical setting based on the results obtained in this study.

## Conclusion

This study showed a strong relationship between EPT and CS, and the prevalence of EPT and AALTF by age in Thailand was first reported. We believe that it helps to know the pathogenesis and biomechanism of EPT and AALTF.

## Data Availability

No datasets were generated or analysed during the current study.

## Abbreviations

EPT: Enlarged peroneal tubercle
AALTF: accessory anterolateral talar facet
CS: Calcaneal spur
